# Clinical Application of Vision Transformers for Melanoma Classification: A Multi-Dataset Evaluation Study

**DOI:** 10.3390/cancers17213447

**Published:** 2025-10-28

**Authors:** Antony Garcia, Jixing Zhou, Gabriela Pinero-Crespo, Thomas Beachkofsky, Xinming Huang

**Affiliations:** 1 Department of Electrical and Computer Engineering, Worcester Polytechnic Institute, Worcester, MA 01609, USA or antony.garcia@utp.ac.pa (A.G.); jzhou1@wpi.edu (J.Z.); 2 Facultad de Ingeniería Eléctrica, Universidad Tecnológica de Panamá, Avenida Universidad Tecnológica de Panamá, Panamá 0819-0728, Panama; 3 College of Medicine, University of South Florida Morsani, Tampa, FL 33602, USA; gpinerocrespo@usf.edu; 4 Dermatology Service, James A. Haley Veterans Hospital, Tampa, FL 33637, USA; thomas.beachkofsky@va.gov

**Keywords:** melanoma, skin cancer, Vision Transformer, dermoscopy, deep learning, medical imaging

## Abstract

**Background**: Melanoma is one of the most lethal skin cancers, with survival rates largely dependent on early detection, yet diagnosis remains difficult because of its visual similarity to benign nevi. Convolutional neural networks have achieved strong performance in dermoscopic analysis but often depend on fixed input sizes and local features, which can limit generalization. Vision Transformers, which capture global image relationships through self-attention, offer a promising alternative. **Methods**: A ViT-L/16 model was fine-tuned using the ISIC 2019 dataset containing more than 25,000 dermoscopic images. To expand the dataset and balance class representation, synthetic melanoma and nevus images were produced with StyleGAN2-ADA, retaining only high-confidence outputs. Model performance was evaluated on an external biopsy-confirmed dataset (MN187) and compared with CNN baselines (ResNet-152, DenseNet-201, EfficientNet-B7, ConvNeXt-XL, ViT-B/16) and the commercial MoleAnalyzer Pro system using ROC-AUC and DeLong’s test. **Results**: The ViT-L/16 model reached a baseline ROC-AUC of 0.902 on MN187, surpassing all CNN baselines and the MoleAnalyzer Pro system, though the difference was not statistically significant (*p* = 0.07). After adding 46,000 confidence-filtered GAN-generated images, the ROC-AUC increased to 0.915, giving a statistically significant improvement over the commercial MoleAnalyzer Pro system (*p* = 0.032). **Conclusions:** Vision Transformers show strong potential for melanoma classification, especially when combined with GAN-based augmentation, offering advantages in global feature representation and data expansion that support the development of dependable AI-driven clinical decision-support systems.

## Simple Summary

Melanoma is a dangerous skin cancer that can be treated successfully when detected early, but it often looks similar to benign moles, which makes diagnosis difficult. This research uses Vision Transformers to help identify melanoma from skin images. The model was trained with real medical images and additional synthetic ones produced by a Deep Learning algorithm to improve learning. Its performance was tested against several Deep Learning classification models and a commercial diagnostic tool. The Vision Transformer achieved higher accuracy in separating cancerous and non-cancerous lesions. This approach may help doctors make faster and more confident assessments when examining skin images, supporting better detection of melanoma in clinical settings.

## 1. Introduction

Melanoma is a malignant tumor developing from melanocytes, the neural crest-derived cells responsible for melanin production in the epidermis. Unlike non-melanoma skin cancers, melanoma exhibits a high propensity for early invasion and metastasis, making it one of the most lethal forms of skin cancer despite its relatively low incidence rate [1].

The pathogenesis of melanoma is driven by a combination of environmental exposures and genetic alterations. Prominent driver mutations include activating alterations in the BRAF gene, with the V600E mutation detected in approximately 40–60% of cutaneous melanomas, along with NRAS (15–20%) and NF1 mutations (10–15%) [1,2,3]. These mutations activate the MAPK and PI3K/AKT pathways, promoting uncontrolled cell proliferation, survival, and migration. In addition, inactivating mutations in tumor suppressor genes like CDKN2A contribute to melanoma progression [4,5]. Intermittent intense sun exposure and other ultraviolet radiation sources induce DNA damage and mutagenesis, acting as a primary environmental initiator in susceptible individuals [6].

Globally, the incidence of melanoma has been rising, especially in fair-skinned populations. According to international epidemiological data, melanoma accounts for approximately 1–2% of all cancer diagnoses worldwide, with over 325,000 new cases and 57,000 deaths annually [7]. It ranks as one of the top five cancers in countries with high UV exposure, such as Australia, New Zealand, and parts of North America and Europe. The increasing incidence among younger individuals, combined with the rising cost of advanced immunotherapies and targeted therapies, contributes to the growing global burden of melanoma [8,9].

Melanoma often appears similar to a harmless mole (nevus), making early detection difficult. This challenge arises because certain benign lesions, such as dysplastic or Spitz nevi, share dermoscopic and structural features with early melanomas [10,11]. The clinical and dermoscopic variability among melanoma subtypes, including acral lentiginous, nodular, superficial spreading, and in situ forms, further complicates early diagnosis [12]. As a result, early-stage tumors are often overlooked or mistaken for benign growths, especially when they lack clear asymmetry or pigment irregularity.

Early-stage detection improves prognosis. Localized melanomas have a 5-year survival rate that exceeds 98%, while metastatic melanoma has a survival rate below 30% despite modern therapies [1]. Thus, improving early diagnostic accuracy remains a priority for reducing melanoma-related mortality. Advances in digital pathology, molecular biomarkers, and AI-assisted dermoscopy are being investigated to improve differentiation between benign and malignant lesions [4,13].

Among AI-assisted tools, convolutional neural networks (CNNs) have been reported to achieve good performance in classifying skin lesions with accuracy comparable to that of dermatologists [14,15,16,17,18,19].

CNN-based models have shown good performance in various clinical settings: Brinker et al. reported that a ResNet-50 CNN outperformed 136 of 157 dermatologists (86%) [15], while Haenssle et al. found that their Inception v4 CNN surpassed most of the 58 dermatologists in diagnostic accuracy [18]. Similarly, Tschandl et al. showed that CNN algorithms significantly outperformed 511 human readers, including 27 experts, in classifying pigmented skin lesions [20]. Only a small minority of dermatologists achieved performance comparable to or better than AI systems, typically fewer than 15–25% of participants. More recent architectures have maintained this trend: Yan et al. showed that their Vision Transformer-based model PanDerm outperformed clinicians by 10.2% in early melanoma detection and achieved diagnostic accuracy comparable to clinicians even without human assistance [21].

However, human–AI collaboration has emerged as the most effective approach, surpassing either modality alone. Hekler et al. showed that combining CNN predictions with dermatologist assessments using a fusion model achieved 89% sensitivity, outperforming both dermatologists alone (66%) and the CNN alone (86.1%) [22]. Tschandl et al. found that AI-based decision support improved human diagnostic accuracy by 13.3%, with the greatest benefits seen in less experienced clinicians [23]. Winkler et al. reported that dermatologists cooperating with a market-approved CNN achieved 100% sensitivity and 86.4% accuracy, significantly exceeding dermatologists alone (84.2% sensitivity, 74.1% accuracy) and CNN alone (81.6% sensitivity, 87.7% accuracy) [24]. In a randomized controlled trial, Han et al. showed that CNN assistance significantly improved nondermatology trainees’ accuracy from 29.7% to 54.7%, though dermatology residents did not show significant improvement [25].

CNNs process dermoscopic images to identify subtle malignancy indicators such as asymmetry, border irregularity, color variation, and diameter [26,27,28,29]. Transfer learning is frequently used in this domain, where models pre-trained on large datasets like ImageNet are fine-tuned on smaller, domain-specific datasets [30,31,32,33]. Emerging approaches using spectral enhancement and multimodal imaging have been explored to improve the representation of diagnostically relevant features [34].

On the other hand, CNNs have structural limitations when applied to diverse populations and imaging conditions. Their architecture relies on spatial locality through convolutional kernels, which restricts their ability to model long-range relationships between distant regions of a lesion and reduces adaptability to variations in imaging devices or illumination. The need for a fixed input size further limits flexibility and can result in the loss of important information during image resizing or cropping. These factors may lead to ignoring delicate dermoscopic textures, such as irregular pigment networks and subtle color gradients extending over multiple spatial scales. In addition, dermoscopic patterns such as regression areas and perifollicular pigmentation often appear in separate regions of an image, requiring analytical models capable of integrating information from distant areas rather than relying solely on local texture patterns [35,36]. Similar limitations have been noted in medical image segmentation studies, where convolutional layers struggle to capture long-range dependencies [37], leading to the development of hybrid CNN–Transformer architectures designed to address these constraints [38].

To address these architectural constraints, Vision Transformers (ViTs) have been introduced as an alternative for image analysis in medicine [39,40]. ViTs apply multi-head self-attention mechanisms that compute pairwise relations among image patches, allowing the model to capture broad contextual structure while maintaining fine detail. Their self-attention mechanism allows the model to capture global contextual dependencies that extend beyond the local neighborhood of pixels, which is valuable in dermoscopic images where diagnostically relevant cues such as pigment networks, vascular structures, and streaks are spatially scattered [36,41].

Unlike CNNs, which rely on local receptive fields, ViTs model global relationships between image patches, preserving fine-grained texture information that CNNs may lose during resizing or pooling of high-resolution images. In addition to modeling global structure, attention-based representations depend less on local pixel statistics that vary with acquisition settings and therefore adapt better when annotated data are limited or originate from heterogeneous clinical sources. Recent studies on ViTs show improved consistency among datasets on several medical imaging benchmarks, including dermatology datasets [35,36,42]. These properties make Vision Transformers appropriate for dermoscopic analysis, where diagnostic cues are subtle, spatially dispersed, and sensitive to imaging variability [43].

ViTs typically require large amounts of training data due to their high parameter count and the absence of inductive biases found in CNNs. This demand creates challenges in medical domains where annotated datasets are often limited. Although large datasets for skin lesions are publicly available, such as the ISIC archive [44], the number of images, which is on the order of tens of thousands, remains small compared with general purpose classification training datasets like ImageNet that contain millions of instances. To address this limitation, data augmentation techniques can be applied. A popular technique involves using Generative Adversarial Networks (GANs) to generate synthetic images or applying self-supervised learning to make use of unlabeled data [45,46,47]. In addition, transfer learning from large-scale pre-trained ViT models offers a practical strategy to reduce the impact of data scarcity. Motivated by these advances, this study applies a Vision Transformer architecture for melanoma classification and compares its performance with that of a widely used commercial system deployed in clinical practice.

In this study, a pretrained ViT model is evaluated to classify dermoscopic images into benign and malignant categories. The model is trained and fine-tuned on the ISIC 2019 dataset, which offers a large collection of high-quality dermoscopic images with expert-provided annotations. To improve the training process, a GAN-based data augmentation pipeline is implemented, generating synthetic images that strengthen the model’s generalization capacity.

The performance of the resulting model was assessed on a new dataset containing images from hospitals in the United States. The images in this dataset received initial classifications from the FotoFinder AI MoleAnalyzer Pro (version 230814_3; FotoFinder Systems GmbH, Bad Birnbach, Germany), a commercial diagnostic tool, and were later confirmed by biopsy. The FotoFinder system is a well-established tool in the domain of skin lesion image classification, as documented in several studies [24,48,49,50,51,52,53,54].

This article is structured as follows. Section 2 describes the experimental setup, including datasets, model architectures, training, augmentation, and evaluation. Section 3 presents the performance results, statistical tests, and the effect of synthetic data on the evaluated models. Section 4 discusses the architectural and clinical implications of the results. Finally, Section 5 summarizes the study, acknowledges its limitations, and outlines directions for future research.

## 2. Materials and Methods

### 2.1. Dataset Preparation

For training the Vision Transformer model in melanoma classification, the ISIC 2019 Challenge dataset was used. This dataset is a large and diverse collection of dermoscopic images compiled by the International Skin Imaging Collaboration [55]. It contains more than 25,000 high-resolution images of skin lesions annotated by expert dermatologists. The collection includes various skin conditions such as benign nevi, dysplastic nevi, and malignant melanomas, covering a broad range of clinical appearances.

The dataset was divided into training and validation, with an external test set. ISIC provides an official train and validation split, with the original ISIC-2019 test set repurposed as the validation set in this study. The training set contained 12,875 nevus images and 4522 melanoma images, while the validation set contained 2495 nevus images and 1327 melanoma images. To evaluate the model’s generalization to unseen clinical data, a new dataset, MN187, was used as the test set.

The MN187 dataset consisted of 187 de-identified dermoscopic images obtained from the VA dermatology clinic (College of Medicine, University of South Florida). It included 96 benign nevi and 91 malignant melanomas, all biopsy confirmed. Each image was initially analyzed by the FotoFinder AI MoleAnalyzer Pro, which produced preliminary malignancy probability scores used for comparative evaluation.

The FotoFinder MoleAnalyzer Pro is a commercially approved CNN-based software for skin lesion classification. It was the first AI application for dermatology to receive approval in the European market [54]. This system uses deep learning algorithms to analyze dermoscopic images and provide malignancy risk assessments with probability scores of 0.0 to 1.0, where higher scores indicate a higher risk of malignancy [49]. The system can identify melanoma, basal cell carcinoma, and most squamous cell carcinomas, including actinic keratoses [50].

Evaluations of the MoleAnalyzer Pro in multiple clinical studies have produced variable outcomes, influenced by the study setting and population. In controlled clinical environments, the system has been reported to have high diagnostic accuracy, with sensitivities from 81.6% to 97.1% and specificities from 78.8% to 94.0% [53]. One prospective diagnostic accuracy study found a sensitivity of 88.1% and specificity of 78.8%, with performance similar to that of expert dermatologists [52]. Another study found that the AI’s diagnostic accuracy was similar to experienced dermatologists, achieving an area under the ROC curve of 0.702 [50].

In contrast, real-world clinical studies have reported more modest results. One study of a high-risk melanoma population found lower metrics, with a sensitivity of 70% and specificity of 40% compared to histopathology as ground truth (ROC-AUC of 0.68) [49]. Other research found even lower performance, with a sensitivity of 36.4% and specificity of 42.1%, and concluded that the system cannot take the place of dermatologist decision-making [48].

The images used in this study were resized and padded to a consistent size of 224 × 224 pixels, which is the input size required by most pre-trained CNN backbone architectures. Padding with black pixels was applied to make images square, which allows us to keep the original proportions of images within the padding and to eliminate distortions that could affect model performance. Although ViT models can handle variable input sizes, input dimensions were standardized in every model to enable fair comparisons.

### 2.2. Model Architecture

Modern deep learning classification models typically consist of a pretrained feature extraction backbone paired with a classification head composed of fully connected layers. The backbone, often a convolutional neural network or a transformer-based model, is trained on large datasets like ImageNet, while the classification head is fine-tuned for the specific task.

In this study, the Vision Transformer was chosen as the backbone architecture. ViTs represent a significant advancement in image classification by using self-attention mechanisms to capture global context. Introduced by Vaswani et al. (2017), attention mechanisms have revolutionized natural language processing and generative AI [56]. Although initially developed for text data, this architecture has been successfully adapted for image analysis [57].

**Figure 1:**
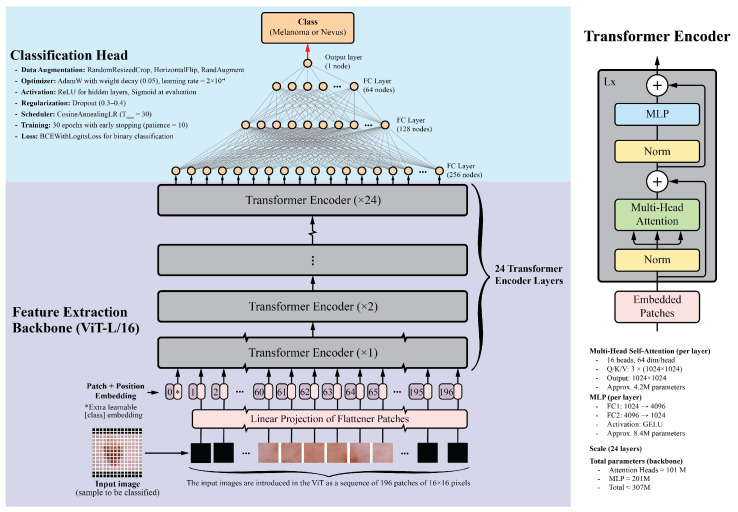
Overview of the Vision Transformer architecture. The input image is divided into patches, which are linearly embedded and passed through transformer layers. The output is a classification token used for final predictions. This diagram was adapted from the original Vision Transformer paper [57].

Vision Transformers divide images into fixed-size patches, embed them linearly, and process them through transformer encoders. This allows ViTs to capture long-range dependencies and complex relationships between image regions. For this study, the ViT-L/16 model pretrained on the ImageNet-1k dataset was selected. This model is one of the largest publicly available pretrained Vision Transformers in the PyTorch library. It contains 24 transformer layers, each with 16 attention heads and a hidden size of 1024. Input images are divided into 16×16 pixel patches, producing a sequence of 196 patches for a standard 224×224 image. Each patch is embedded into a 1024-dimensional vector, with positional embeddings added to preserve spatial information. The transformer layers include multi-head self-attention mechanisms, feed-forward neural networks, layer normalization, and residual connections. With approximately 307 million parameters, the detailed architecture and implementation specifics, including the learning rate schedule, optimizer settings, dropout regularization, data augmentation, and training epochs, are summarized in Figure 1.

The classification head consists of four fully connected layers with ReLU activations: 256, 128, 64, and 1 neurons, respectively. The final layer uses a sigmoid activation function to generate a probability score between 0 and 1, indicating the probability of malignancy. Dropout layers with a 0.5 dropout rate were added after each fully connected layer to prevent overfitting.

For comparison, several CNN backbones were evaluated, including ResNet-152, DenseNet-201, EfficientNet-B7, and InceptionV4. These models, recognized as top performers in their respective architecture families, have achieved good results in image classification tasks, including skin lesion analysis [17,58,59,60]. Additionally, the ViT-B/16 model, a smaller variant of the ViT-L/16, was included. This model features 12 transformer layers, 12 attention heads, and a hidden size of 768, while maintaining a similar architecture to that shown in Figure 1. The classification head architecture was the same for all models to ensure a fair comparison.

### 2.3. Training Configuration and Optimization

All experiments were implemented in PyTorch (version 2.8.0) and executed on a pair of NVIDIA RTX A5000 GPUs with 48 GB total VRAM. For consistency among models, a unified training configuration was adopted. Images were normalized using the ImageNet mean and standard deviation ([0.485, 0.456, 0.406], [0.229, 0.224, 0.225]), in line with the pretrained weights. The training pipeline included random resized cropping to 224 pixels with a scale range of 0.7 to 1.0, random horizontal flipping, and RandAugment. The training set was loaded with a batch size of 128 and shuffled at each epoch, while the validation and test sets were processed with batch sizes of 64 images.

The Vision Transformer backbone was initialized with ImageNet-1k pretrained weights. The transformer backbone parameters were frozen, and only the classification head was trained.

Training optimization used binary cross-entropy with logits (BCEWithLogitsLoss) as the loss function. Parameters were updated with the AdamW optimizer configured with a learning rate of $2\times 10^{-4}$ and a weight decay of 0.05. A cosine annealing learning rate scheduler with $T_{\max}=30$ progressively reduced the learning rate through epochs.

### 2.4. Data Augmentation

To address class imbalance and broaden the training distribution, generative models were applied to the ISIC 2019 challenge dataset, which contains 12,875 nevus and 4522 melanoma images. Separate GAN models were trained for each class on images and resized to 256×256 pixels, allowing the networks to capture class-specific distributions.

Table 1 lists the principal parameters and settings used for the StyleGAN2-ADA models. The table summarizes hardware configuration, dataset preprocessing, optimizer setup, and transfer learning details applied during fine-tuning from a pretrained checkpoint.

The GAN models followed the StyleGAN2-ADA architecture described in [61] and used the official NVIDIA implementation [62], with modifications introduced for compatibility with newer PyTorch and CUDA versions. The StyleGAN2-ADA training procedure is based on *ticks*, where each tick processes a fixed number of images. Each model was trained for 1000 ticks, and snapshots were recorded every five ticks to monitor generator progress.

The FID50k_full metric, which computes the Fréchet Inception Distance (FID) over 50,000 generated images, served to monitor convergence. Each GAN required approximately two to three days of training under these conditions.

After training, each GAN produced 25,000 images for its class, resulting in a total of 50,000 synthetic samples. These generated images maintained consistency with real dermoscopic data and reflected intra-class variability. Generated outputs were filtered using the ViT-L/16 classifier to exclude samples with low confidence or unrealistic appearance before inclusion in the augmented dataset. This filtering step was applied only to synthetic images derived from the ISIC 2019 dataset. No data from the MN187 set were used, referenced, or accessed during GAN training or filtering, and MN187 remained fully reserved for final evaluation.

All synthetic images were resized and normalized to match the preprocessing of the real data (224×224 pixels, normalized with ImageNet statistics). The combined set of real and generated images formed a larger and more balanced training dataset that improved the performance of the classification model.

### 2.5. Model Performance Monitoring and Evaluation

The Receiver Operating Characteristic Area Under the Curve (ROC-AUC) serves as the primary evaluation metric in this study. This threshold-independent metric measures binary classification performance by quantifying the model’s capacity to separate positive and negative classes through all classification thresholds [63,64,65]. ROC-AUC is appropriate for imbalanced datasets and finds common application in medical imaging tasks such as skin lesion classification.

A high ROC-AUC score alone does not fully characterize model performance. For instance, a model might obtain a high score by correctly ranking a small number of straightforward cases, which could overstate its general discriminative capability [66].

To evaluate the statistical significance of performance differences, the ROC-AUC of each model was compared with a predefined baseline model using DeLong’s test [67]. This nonparametric method is a well-established approach for comparing the AUCs of two classifiers tested on the same dataset, accounting for the correlated nature of their predictions and the variability among paired samples.

The statistical hypotheses were defined as follows: the null hypothesis ($H_{0}$) states that no difference in ROC-AUC exists between the baseline model and the evaluated model ($AUC_{\mathrm{baseline}}=AUC_{\mathrm{evaluated}}$); the alternative hypothesis ($H_{1}$) states that a difference exists ($AUC_{\mathrm{baseline}}\neq AUC_{\mathrm{evaluated}}$). A significance level of α=0.05 was applied uniformly to all comparisons, and 95% confidence intervals were estimated to quantify the uncertainty associated with each AUC difference. A *p*-value from DeLong’s test below α (p<0.05) was interpreted as evidence of a statistically significant difference in model performance, whereas values of p≥0.05 were considered consistent with random variation, implying no significant difference between models.

### 2.6. Model Interpretability

To improve the interpretability of the Vision Transformer–based skin lesion classifier, an attention-based visualization technique was applied to make explicit how the model distributes its focus across image regions during prediction. This approach provides a clear view of the spatial behavior of the network by presenting the self-attention responses computed within the transformer encoder.

In the ViT architecture, each input image $I\in R^{H\times W\times 3}$ is divided into a sequence of *N* non-overlapping patches $\left\{x_{1},x_{2},\ldots ,x_{N}\right\}$, each projected into an *D*-dimensional embedding space [57]. Within each transformer layer *l*, the self-attention mechanism evaluates pairwise relationships among all patch tokens. For a given attention head *h*, the attention scores are computed as formulated in [56]:

$$A^{\left(l,h\right)}=\mathrm{softmax}\;\left(\frac{Q^{\left(l,h\right)}{\left(K^{\left(l,h\right)}\right)}^{⊤}}{\sqrt{d_{k}}}\right),$$

where $Q^{\left(l,h\right)}=X^{\left(l\right)}W_{Q}^{\left(l,h\right)}$, $K^{\left(l,h\right)}=X^{\left(l\right)}W_{K}^{\left(l,h\right)}$, and $X^{\left(l\right)}$ denote the input patch embeddings to layer *l*. The resulting attention matrix $A^{\left(l,h\right)}\in R^{N\times N}$ represents the strength of interaction between image patches.

A single attention map per layer is obtained by averaging the attention matrices among all heads:

$$A^{\left(l\right)}=\frac{1}{H}\sum_{h=1}^{H}A^{\left(l,h\right)},$$

where *H* is the number of attention heads. The first row of $A^{\left(l\right)}$, corresponding to the [CLS] token, is taken as the measure of global attention assigned to each image patch. This attention is reshaped into a spatial grid and displayed as a heatmap, with higher intensity values representing greater relevance to the model’s decision [68].

Through this process, the evolution of the model’s focus through transformer layers can be examined, from diffuse attention in the earlier stages to more spatially localized activation patterns in deeper layers. Examination of these attention distributions helps determine of whether the model concentrates on clinically relevant regions in the dermoscopic images, thus supporting interpretability in its diagnostic behavior [69].

## 3. Results

### 3.1. Model Performance

The proposed models were evaluated using the ROC-AUC metric. Table 2 summarizes the results for six deep learning classification models, each using distinct backbones for feature extraction while sharing the same classification head, as depicted in Figure 1. These models were trained on the ISIC-19 dataset, using standard image transformations (Resize, CenterCrop, and Normalization) integrated into the training pipeline. Validation was performed on the ISIC-19 dataset, and testing was performed on the MN187 dataset.

**Figure 2:**
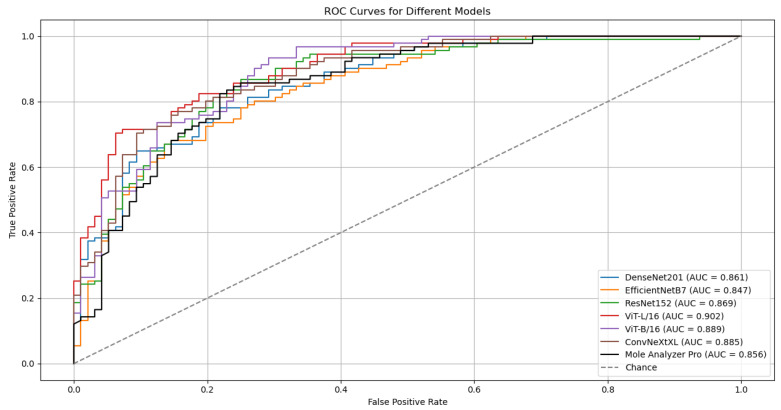
ROC curves and AUC scores for the ViT-L/16 model on the validation (ISIC-19) and test (MN187) datasets.

The outcomes of six runs for each model are detailed in Table 2. The ROC Curves and AUC scores for the top-performing model, ViT-L/16, on both validation and test datasets are presented in Figure 2.

Although the models were trained on the ISIC-19 dataset, they kept generalization capabilities when evaluated on the MN187 dataset, performing at a level similar to or exceeding the MoleAnalyzer Pro. The ViT-L/16 model achieved the highest ROC-AUC score of 0.902 on the MN187 dataset, exceeding all other models. The ConvNeXt-XL, a CNN-based model larger than ViT-L/16 in terms of parameters, achieved the second-highest ROC-AUC score of 0.885, comparable to the ViT-B/16 model. The other CNN models had lower performance, aligning more closely with the MoleAnalyzer Pro.

While most models achieved higher ROC-AUC scores than the MoleAnalyzer Pro, the statistical significance of these improvements was evaluated using DeLong’s test. Table 3 summarizes the results, comparing the ROC-AUC of each deep learning model to that of the MoleAnalyzer Pro.

The ViT-L/16 model achieved an ROC-AUC of 0.901, which exceeds the MoleAnalyzer Pro ROC-AUC of 0.856. However, the *p*-value of 0.070 means this difference is not statistically significant at the 0.05 significance level. The CNN models (EfficientNet-B7, DenseNet-201, ConvNeXt-XL) and the ViT-B/16 also had higher ROC-AUC scores than the MoleAnalyzer Pro, but their *p*-values suggest these differences are not statistically significant. None of the models had a performance improvement that could be attributed to factors other than chance, so the null hypothesis could not be rejected.

### 3.2. Results with Data Augmentation

While models like ViT-L/16 and ConvNeXt-XL achieved higher ROC-AUC scores and lower probabilities of the differences being due to chance, they did not reach a statistically significant improvement over the MoleAnalyzer Pro. Vision Transformers, due to their high parameter count and lack of inherent CNN inductive biases, often need large volumes of training data for optimal performance.

**Figure 3:**
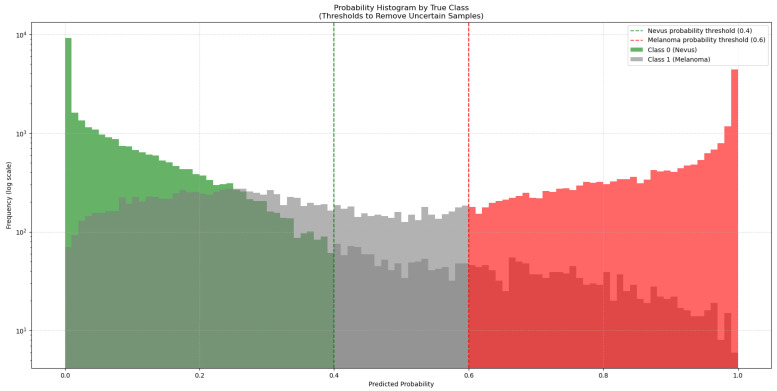
Distribution of predicted probabilities for the synthetic images generated by the GAN models. The green bars represent synthetic nevus images, while the red bars represent synthetic melanoma images. Gray bars represent uncertain classifications. The vertical dashed lines mark the thresholds used to select high-confidence synthetic images for training.

To address this, 50,000 synthetic images generated with GANs were added to the training dataset. As these synthetic images come from a trained GAN model, they might not match the true data distribution perfectly and could contain artifacts or biases. To manage this, the synthetic images were classified by the ViT-L/16 model, which had the highest ROC-AUC score. This model generated a new subset from the 50,000 synthetic images by choosing only those it classified with high confidence (probability > 0.6 for melanoma and <0.4 for nevus), as seen in Figure 3. This method added only high-quality synthetic images that were close to real images to the training set.

**Figure 4:**
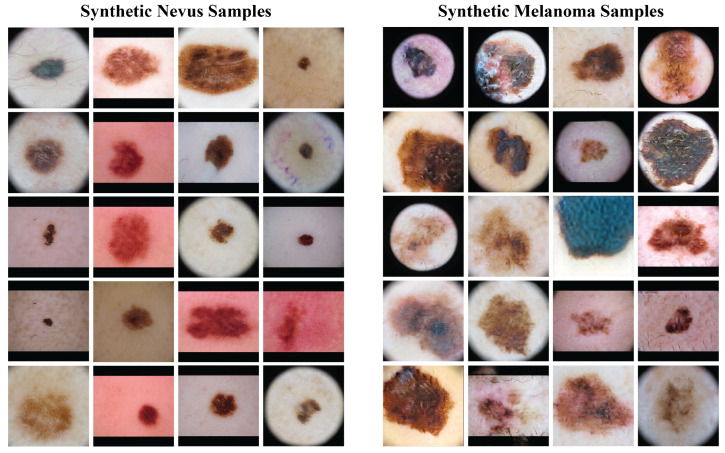
Samples of synthetic images generated by the GAN models. The **left** column shows synthetic nevus images, while the **right** column shows synthetic melanoma images.

Figure 4 presents a sample of the synthetic images from the GANs. The left column has synthetic nevus images, and the right column has synthetic melanoma images. These images have a range of colors, textures, and patterns found in real dermoscopic images. The GANs produced realistic images that could be useful for training the classification model.

Before the addition of synthetic images, the ISIC-19 dataset had 12,875 nevus and 4522 melanoma images (class proportions of 74.0% nevus and 26.0% melanoma). After filtering with the ViT-L/16 model, 27,793 nevus and 18,438 melanoma synthetic images were chosen. This created a new training dataset with a total of 40,668 nevus and 22,960 melanoma images (adjusting the class proportions to 63.9% nevus and 36.1% melanoma), which increased the size and diversity of the training data.

### 3.3. Model Performance with Data Augmentation

Including high-confidence synthetic images in the training data produced performance gains for all model architectures when compared to their performance without synthetic data (presented in Table 2). Table 4 presents results from six independent training runs on both the validation (ISIC-2019) and external test (MN187) sets. The uniformity of these improvements over multiple runs supports the conclusion that the data augmentation strategy provided a general benefit.

**Figure 5:**
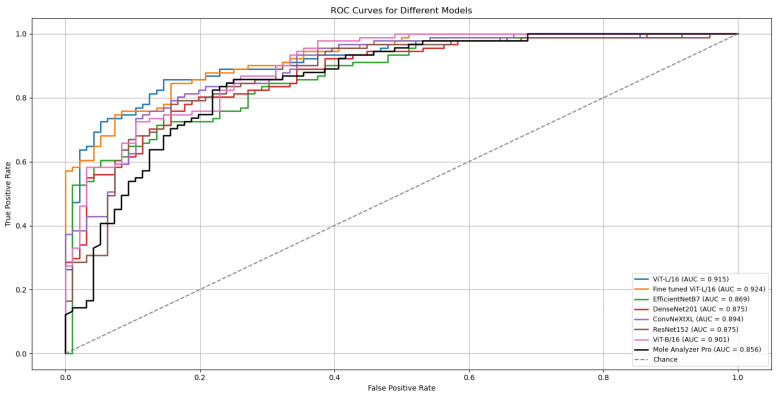
ROC curves and AUC scores for the multiple deep learning models trained with confidence-filtered GAN-augmented data on the validation (ISIC-2019) and external test (MN187) datasets.

The ViT-L/16 architecture remained the strongest performer, reaching a top ROC-AUC of 0.915 on the challenging MN187 test set, as presented in Figure 5. This result exceeds its previous best score of 0.902. Other models also showed classification performance improvements; the ConvNeXt-XL model advanced from a previous score of 0.885 to 0.894. Similar positive trends were observed for the ViT-B/16, ResNet-152, EfficientNet-B7, and DenseNet-201 models, confirming that the augmented dataset created a more effective training environment.

To evaluate the statistical significance of these improvements, the best run from each model was compared against the established benchmark, MoleAnalyzer Pro, using DeLong’s test. The results of this analysis are in Table 5. The standard ViT-L/16 model, with an AUC of 0.915, achieved a statistically significant advantage over the commercial software (*p* = 0.032).

All statistical tests were performed relative to the commercial MoleAnalyzer Pro system. No statistical comparison was conducted between the augmented and non-augmented ViT-L/16 models, as the focus was on demonstrating whether augmentation resulted in statistically validated performance relative to the clinical benchmark.

A refined version of the ViT-L/16 model was also developed for further improvement. This fine-tuned model, included in Figure 5 and Table 5, was created by initializing with the best ViT-L/16 run, freezing its classification head, and unfreezing the final six transformer layers. These layers were then trained for an additional 30 epochs with a reduced learning rate (1 × 10^−4^).

The model was trained on the same ISIC 2019 dataset used in the previous runs that optimized only the classification head, but this time, the focus was on updating the final six transformer layers of the encoder. Training followed the same structure as previous ViT runs, using identical data splits, augmentations, and optimization parameters. The AdamW optimizer with cosine annealing and a weight decay of 0.05 was applied, along with early stopping (patience = 15).

This stage began from the previously best-performing pretrained model, using the same learned weights as initialization. The earlier parameters were frozen to preserve their representations, and training was restarted with updates restricted to the last six of the 24 transformer layers. This design allowed the model to adjust its higher-level representations to the ISIC 2019 domain while maintaining stable lower-level features.

The outcome was a model that achieved an ROC-AUC of 0.926 on the MN187 test set. The statistical comparison with MoleAnalyzer Pro for this model produced a *p*-value of 0.006, providing evidence that its improved performance is statistically significant.

### 3.4. Attention Map Analysis

Attention maps from all 24 layers of the Vision Transformer were analyzed to observe how the model’s focus changed through the layers. In the initial layers, attention was distributed between both the lesion and the surrounding background, capturing structural features like overall shape, border contrast, and color patterns, similar to how a clinician begins a visual assessment.

In deeper layers, attention became more concentrated within smaller regions of the image, often centered on irregular pigment clusters or darker zones. This progression shows how the Vision Transformer gradually shifts from processing general spatial information to concentrating on detailed features that support accurate classification.

**Figure 6:**
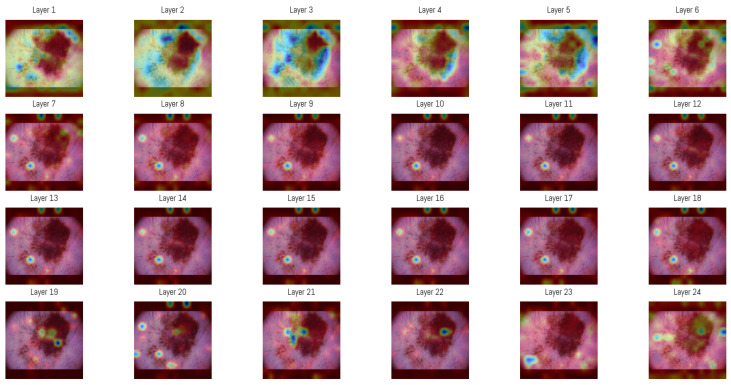
Attention maps in each of the 24 layers of the Vision Transformer model. The sequence of maps shows a transition from broad and diffuse attention patterns in the early layers to more specific regions in the later stages of processing. In these visualizations, darker blue regions represent the highest attention scores in each layer, while colors shifting toward green and yellow indicate progressively lower attention scores. Areas without color correspond to regions where little or no attention is assigned.

For visualization, the attention matrices from each layer were averaged among all heads and represented as heatmaps (Figure 6). This was generated by selecting one sample from the MN187 dataset and using the best-performing, fine-tuned Vision Transformer model (AUC 0.926). As shown, the attention maps evolve from broadly distributed patterns in the initial layers to more concentrated activations in the deeper layers.

An averaged attention map was produced by combining the responses from layers 3 to 5 (Figure 6). The overlaid heatmap representations were derived from the attention tensors of these layers, normalized across layers 3, 4, and 5, then averaged and rendered as a single heatmap. The choice of layers was guided by an entropy-based analysis performed on the validation subset of the ISIC dataset. Entropy was computed from the same attention tensors to measure the variability of activation among image regions, and the mean entropy per layer was obtained by averaging over all samples. Layers 3–5 showed the highest mean entropy values among the 24 layers, presenting greater diversity in spatial representations. The corresponding entropy values for all layers are presented in Table 6.

**Figure 7:**
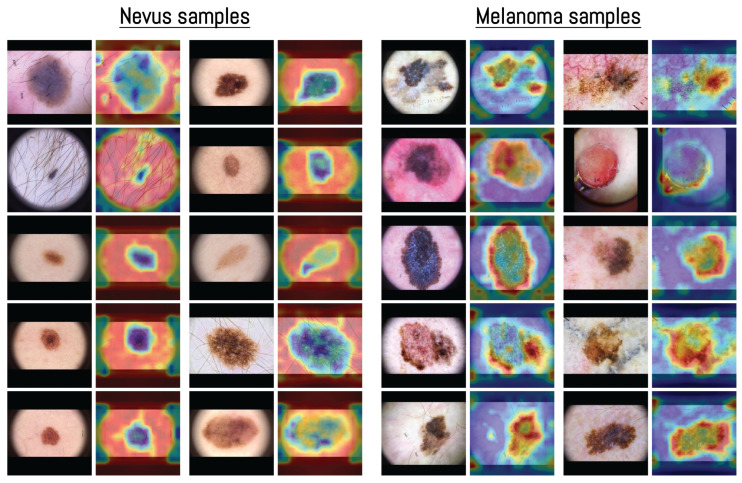
Examples of averaged attention maps obtained from layers 3–5 of the Vision Transformer model. Each pair of images shows the original dermoscopic input (**left**) and its corresponding attention-based visualization (**right**). Samples are organized by class to present the distribution of attention across different inputs. The color scale was adjusted according to the classification score, with images predicted as nevus displayed in blue surrounded by red, and those predicted as melanoma displayed in red surrounded by blue. All samples were selected from the MN187 dermoscopic dataset.

The visualization in Figure 7 shows how the model distributed its spatial attention. In several nevus examples, attention was spread across outer structures such as terminal hairs around the lesion, shown as darker blue areas in the heatmap. For instance, in the first nevus image, the model focused more on the thin terminal hairs extending from the lesion surface rather than the central pigmented region, indicating that these outer features were included in the model’s internal representation. This pattern of attention related to terminal hairs is consistent with dermoscopic observations, where hair growth within a lesion is often associated with benign nevi [70].

In contrast, melanoma samples often showed attention focused along the lesion borders or around uneven outer regions. This pattern matches known dermoscopic features such as irregular borders and uneven pigment distribution [71]. Some nevus heatmaps showed broader attention covering most of the lesion, while melanoma heatmaps concentrated on specific irregular areas or edges, suggesting that the model captured spatial patterns similar to those used in clinical dermoscopic assessment.

## 4. Discussion

The results of this study provide evidence for the potential of Vision Transformers in melanoma classification using dermoscopic images. The ViT-L/16 model achieved the highest ROC-AUC score among all evaluated models, which demonstrates its capacity to capture global contextual information in dermoscopic images. This performance supports the advantages of self-attention mechanisms when analyzing complex visual patterns found in skin lesions.

Despite promising results, the ROC-AUC differences compared with MoleAnalyzer Pro were not statistically significant in the initial experiments. This result shows the difficulty of achieving measurable performance gains in medical imaging, where high baselines and limited datasets restrict model development. Adding synthetic images produced by a GAN addressed data scarcity and produced a statistically significant improvement when compared with the commercial MoleAnalyzer Pro system (*p* = 0.032). This result implies that data augmentation can improve model generalization in domains with limited annotated data.

Using a GAN to generate synthetic images provided a practical approach to augment the training dataset. Filtering synthetic images based on model confidence reduced the chance of introducing artifacts or biases. This selection process limited the set to higher-quality synthetic images, which likely improved ViT-L/16 performance. However, reliance on synthetic data also raises concerns about possible limitations, including the risk of overfitting to the synthetic distribution.

When comparing ViT and CNN architectures, ViT generally outperformed the CNN-based models in this study. Although model size is an important factor, the superior performance of ViT-L/16 cannot be explained by parameter count alone. For example, the CNN-based ConvNeXt Large model contains more parameters than ViT-L/16 yet achieves lower performance. The results support the view that the Transformer architecture itself, with its self-attention mechanism, may be a better suited for the task. This result is consistent with recent reports showing stronger outcomes from ViT on a range of medical imaging applications [35,36,42]. However, ViT models require greater computational resources during both training and inference, which can limit their practicality in resource-constrained settings.

Another important point is the need for statistical validation when assessing model performance. Although ViT-L/16 produced higher ROC-AUC scores than MoleAnalyzer Pro, the absence of statistical significance in the initial comparison highlights the need for tests such as DeLong’s method to verify that the observed gains are not due to chance. In this study, all statistical comparisons were performed relative to the commercial MoleAnalyzer Pro system to determine whether the proposed models reached clinically validated performance levels. In clinical research, reporting metrics without such validation can be misleading, since a numerically higher value may not correspond to a real advancement. For applications that influence medical decisions, establishing statistical significance is an important step toward building confidence in a model’s reported results. This practice helps to ensure that performance claims are reliable and not simply the result of variability in the training process.

The fine-tuned ViT-L/16 variant, obtained by unfreezing only the last six transformer blocks and training them with a reduced learning rate, delivered an incremental improvement (ROC-AUC 0.926; *p* = 0.006 vs. MoleAnalyzer Pro) over the frozen-backbone configuration (best ROC-AUC 0.915). This suggests that limited, targeted adaptation of high-level self-attention layers helps align global contextual features with dermoscopic lesion morphology without incurring instability or overfitting. Expanding fine-tuning depth, applying layer-wise learning rate decay, or integrating lightweight adapters (such as LoRA) may further improve performance, but these strategies can lead to an increased risk of overfitting in limited-data and domain-shift settings [72,73,74].

Beyond performance, the Vision Transformer handles interpretability differently from convolution-based systems such as MoleAnalyzer Pro. Its self-attention layers produce maps showing which image regions contribute to the classification result, as described in the results. In dermoscopic images, these regions often align with visible characteristics such as uneven pigmentation, irregular borders, or streaks near the lesion edge. CNN-based approaches like Grad-CAM rely on gradient information to visualize relevant areas. The attention structure in Transformers provides an alternative representation, based on spatial relations within the model, which can be examined to see how attention is distributed during prediction and whether it corresponds to features used in dermatologic assessment.

Commercial skin analysis platforms such as MoleAnalyzer Pro, which currently rely on convolutional neural networks, could benefit from the integration of Vision Transformer models. These newer models generalize better, providing accurate diagnoses on data not seen during training. An important architectural advantage is their flexibility with input sizes, which removes the need for preprocessing steps such as padding and resizing. This combination of properties reduces manual image tuning and leads to more consistent performance in practice.

## 5. Conclusions

This study evaluated the clinical potential of Vision Transformers (ViTs) for melanoma classification using two dermoscopic datasets. Within a standardized experimental framework, the ViT-L/16 architecture consistently outperformed conventional CNN-based models and the commercial FotoFinder MoleAnalyzer Pro system. A statistically significant improvement in predictive performance was observed only when the GAN-augmented ViT-L/16 model was compared with MoleAnalyzer Pro (*p* = 0.032). This outcome supports the usefulness of synthetic data in addressing class imbalance and the limited availability of labeled clinical images.

Several conclusions can be drawn. First, ViTs effectively capture global contextual information within dermoscopic images, leading to more accurate separation of malignant and benign lesions. Second, when filtered carefully, GAN-based augmentation improves data diversity and generalization, reducing the constraints imposed by relatively small medical imaging datasets. Third, statistical evaluation with DeLong’s test is necessary to verify that observed performance gains are reproducible and clinically meaningful.

The study also has limitations. The external validation dataset, although biopsy-confirmed, was limited in size and diversity, which may affect general applicability. Furthermore, the analysis focused exclusively on binary classification (melanoma versus nevus), without considering other skin lesion categories. Expanding the approach to multi-class classification and incorporating additional clinical factors could improve practical relevance.

Future work should include larger and more diverse datasets, integration of clinical metadata and patient risk profiles to provide diagnostic context, and the adoption of interpretable AI approaches to strengthen clinician confidence. These steps would support the progression of Vision Transformer–based systems toward real-world dermatology applications and contribute to earlier melanoma detection and better patient outcomes.

## Figures and Tables

**Table 1:** Summary of parameters and configurations used for training the GAN model (StyleGAN2-ADA). The –data file parameter shown in this table is dataset_nevus.zip, which contains the nevus samples used for training. For the melanoma model, a separate dataset_melanoma.zip file was used. Each GAN model required its corresponding training samples to be provided in a separate ZIP file, resulting in independently trained models that generated distinct samples.

Category	Parameter	Value / Setting	Description / Notes
Hardware	–gpus	2	Number of GPUs used (2× NVIDIA RTX A5000)
	–workers	3	Number of DataLoader workers per process
Dataset	–data	dataset_nevus.zip	Path to training dataset (datasets are provided to the model in zip compressed files. Both datasets, nevus and melanoma, are trained with the same parameters, only changing the dataset file)
	–mirror	True	Enables x-flip augmentation (data doubling)
	–subset	None	Uses full dataset (no subset restriction)
	Resolution	256×256	Input image resolution for generator and discriminator
Base Configuration	–cfg	auto	Auto-selects model depth, learning rate, and γ
	–kimg	1000	Total training duration (1000 k images = 1 M samples)
	–batch	16	Global batch size
	–gamma	Auto (≈$0.0002\times {\mathrm{res}}^{2}/\mathrm{batch}$)	R1 regularization weight (≈13.1 at 256×256)
	–snap	5	Snapshot / FID computation interval (in ticks)
	Optimizer	Adam	Used for both Generator and Discriminator
	Metrics	[FID_50*k_full*_]	Frechet Inception Distance computed every snapshot
Augmentation	–aug	noaug	No adaptive discriminator augmentation applied
	–augpipe	Default (bgc)	Pipeline defined but inactive due to noaug
	–target	N/A	ADA target (0.6 default) unused in this configuration
Optimization	Learning rate	0.002–0.0025	Determined automatically by resolution and batch size
	Betas	(0, 0.99)	Adam momentum parameters
	ϵ	1 × 10^−8^	Adam numerical stability constant
	EMA ramp-up	Enabled (10–20 kimg)	Stabilizes generator weights early in training
	Mixed precision	Enabled (FP16)	Reduces GPU memory usage, improves throughput
	TF32 / cuDNN	Enabled	Allows TensorFloat32 for convolutions; benchmarking on
Model Architecture	Latent dimensions	512	StyleGAN2 latent and mapping vector size (*z*, *w*)
	Mapping layers	8	Depth of style mapping network (MLP)
	Channel base / max	32,768/512	Controls feature-map width scaling
	–freezed	None	No frozen discriminator layers
	EMA decay	Auto (0.999–0.9999)	Exponential moving average rate for generator weights
Evaluation	Metric	FID_50*k_full*_	Computed on 50k generated images using Inception-V3
	Stopping criterion	FID < 50	Early stopping once quality threshold achieved

**Table 2:** This table presents the results of running six different deep learning classification models, each using distinct backbones for feature extraction but the same classification head, as illustrated in Figure 1. The validation results are based on the ISIC-19 dataset, while the testing was conducted using the MN187 dataset.

Model	Validation (ISIC-2019)					Testing (MN187)				
	Run 1	Run 2	Run 3	Run 4	Run 5	Run 1	Run 2	Run 3	Run 4	Run 5
**ViT-L/16**	0.921	0.919	0.919	0.920	0.919	0.902	0.896	0.896	0.902	**0.902**
**ViT-B/16**	0.919	0.916	0.915	0.914	0.916	0.887	**0.889**	0.877	0.886	0.882
**ResNet-152**	0.909	0.908	0.907	0.908	0.908	0.857	0.861	**0.869**	0.860	0.861
**EfficientNet-B7**	0.889	0.887	0.887	0.889	0.889	0.847	0.835	0.838	0.834	**0.839**
**DenseNet-201**	0.915	0.916	0.914	0.914	0.914	0.842	0.858	0.855	**0.861**	0.845
**ConvNeXt-XL**	0.926	0.927	0.929	0.926	0.928	0.881	0.877	0.881	0.884	**0.885**

**Table 3:** Summary of DeLong test results comparing model AUCs to the MoleAnalyzer Pro AUC. The *p*-values address whether the differences in AUCs are statistically significant.

Model	Deep Learning Model AUC	MoleAnalyzer Pro AUC	DeLong Test *p*-Value
**ViT-L/16**	0.901	0.856	0.070
**ConvNeXt-XL**	0.884	0.856	0.226
**ViT-B/16**	0.888	0.856	0.240
**ResNet-152**	0.868	0.856	0.654
**EfficientNet-B7**	0.846	0.856	0.709
**DenseNet-201**	0.859	0.856	0.910

**Table 4:** ROC-AUC results (five runs each) for six models trained with the augmented dataset (real ISIC-2019 + confidence-filtered GAN synthetic images). Left block: validation (ISIC-2019). Right block: external test (MN187). Bold values indicate the best test run per model.

Model	Validation (ISIC-2019)					Testing (MN187)				
	Run 1	Run 2	Run 3	Run 4	Run 5	Run 1	Run 2	Run 3	Run 4	Run 5
**ViT-L/16**	0.938	0.936	0.936	0.937	0.935	0.914	0.909	0.908	**0.915**	0.908
**ViT-B/16**	0.924	0.927	0.927	0.928	0.927	0.896	0.892	0.891	**0.901**	0.899
**ResNet-152**	0.911	0.909	0.912	0.912	0.911	**0.875**	0.870	0.873	0.872	0.872
**EfficientNet-B7**	0.895	0.894	0.893	0.894	0.893	0.860	0.866	0.863	0.866	**0.869**
**DenseNet-201**	0.921	0.921	0.920	0.920	0.921	0.870	0.868	0.872	**0.875**	0.861
**ConvNeXt-XL**	0.934	0.936	0.933	0.933	0.934	0.891	**0.894**	0.885	0.889	0.894

**Table 5:** Summary of DeLong test results comparing model AUCs to the MoleAnalyzer Pro AUC. The *p*-values address whether the differences in AUCs are statistically significant.

Model	Deep Learning Model AUC	MoleAnalyzer Pro AUC	DeLong Test *p*-Value
**ViT-L/16**	0.920	0.856	0.032
**Fine tuned ViT-L/16**	0.926	0.856	0.006
**EfficientNet-B7**	0.870	0.856	0.645
**DenseNet-201**	0.880	0.856	0.466
**ConvNeXt-XL**	0.897	0.856	0.154
**ResNet-152**	0.877	0.856	0.498
**ViT-B/16**	0.905	0.856	0.098

**Table 6:** Average entropy ranking of attention layers in the Vision Transformer model. Higher entropy values represent greater diversity in spatial representations.

Rank	Layer	Average Entropy
1	Layer 3	0.8615
2	Layer 4	0.8463
3	Layer 5	0.8393
4	Layer 1	0.8166
5	Layer 6	0.8065
6	Layer 2	0.7685
7	Layer 7	0.7272
8	Layer 24	0.7241
9	Layer 23	0.7036
10	Layer 8	0.6853
11	Layer 9	0.6004
12	Layer 21	0.5624
13	Layer 18	0.5598
14	Layer 17	0.5418
15	Layer 11	0.5405
16	Layer 16	0.5379
17	Layer 13	0.5339
18	Layer 12	0.5331
19	Layer 15	0.5322
20	Layer 10	0.5187
21	Layer 14	0.5141
22	Layer 22	0.4742
23	Layer 20	0.4740
24	Layer 19	0.4354

## Data Availability

The data supporting the findings of this study (including the MN187 clinical dermoscopic images and derived model outputs) are available from the corresponding author upon reasonable request. Due to patient privacy and institutional review constraints, the raw clinical images cannot be placed in a public repository. De-identified metadata, trained model weights, and synthetic GAN-generated images can be shared subject to a data use agreement.
